# Indoleamine 2,3 Dioxygenase 1—The Potential Link between the Innate Immunity and the Ischemia-Reperfusion-Induced Acute Kidney Injury?

**DOI:** 10.3390/ijms23116176

**Published:** 2022-05-31

**Authors:** Anna Krupa, Mikolaj M. Krupa, Krystyna Pawlak

**Affiliations:** 1 Department of Internal Medicine and Metabolic Diseases, Medical University of Bialystok, M. Sklodowskiej-Curie 24A, 15-276 Bialystok, Poland; anna.krupa@umb.edu.pl; 2 Department of Monitored Pharmacotherapy, Medical University of Bialystok, Mickiewicza 2C, 15-222 Bialystok, Poland; 38327@student.umb.edu.pl

**Keywords:** indoleamine 2,3 dioxygenase 1 (IDO1), kynurenine pathway (KP), innate immunity, acute kidney injury (AKI), ischemia-reperfusion injury (IRI)

## Abstract

Ischemia-reperfusion injury (IRI) is of the most common causes of acute kidney injury (AKI); nevertheless, the mechanisms responsible for both early kidney injury and the reparative phase are not fully recognised. The inflammatory response following ischemia is characterised by the crosstalk between cells belonging to the innate immune system—dendritic cells (DCs), macrophages, neutrophils, natural killer (NK) cells, and renal tubular epithelial cells (RTECs). A tough inflammatory response can damage the renal tissue; it may also have a protective effect leading to the repair after IRI. Indoleamine 2,3 dioxygenase 1 (IDO1), the principal enzyme of the kynurenine pathway (KP), has a broad spectrum of immunological activity from stimulation to immunosuppressive activity in inflamed areas. IDO1 expression occurs in cells of the innate immunity and RTECs during IRI, resulting in local tryptophan (TRP) depletion and generation of kynurenines, and both of these mechanisms contribute to the immunosuppressive effect. Nonetheless, it is unknown if the above mechanism can play a harmful or preventive role in IRI-induced AKI. Despite the scarcity of literature in this field, the current review attempts to present a possible role of IDO1 activation in the regulation of the innate immune system in IRI-induced AKI.

## 1. Introduction

Acute kidney injury (AKI) is a clinical syndrome characterised by the deterioration of kidney function, which according to KDIGO, is accompanied by an increased creatinine concentration in serum or a decrease in diuresis [1]. A high level of mortality, the possibility of developing chronic kidney disease, and expensive costs of hospitalisation are just a few features that describe the indicated problems of healthcare systems around the world [2,3].

In the clinical environment, AKI is mainly caused by ischemia-reperfusion injury (IRI), nephrotoxicity, and sepsis; moreover, IRI is considered as a major cause of AKI associated with multiple clinical situations, occurring with renal tissue hypoperfusion [4]. AKI caused by IRI represents a principal clinical problem during the perioperative period, leading to a higher risk of complications and increased mortality [5]. Furthermore, patients who had AKI, even mild or short, tend to have a higher risk of developing chronic kidney disease at later stages of their life [6,7].

IRI is a very complex process, which, in general, consists of two pathophysiologically distinct phases: ischemia and reoxygenation. Several clinical conditions, such as acute coronary syndrome, sepsis, haemorrhage, major surgery, and kidney transplantation may cause kidney tissue hypoperfusion, defined as ischemia. The ischemia leads to dysfunction of the mitochondrial electron transport chain and a decrease in ATP synthesis in those organelles. The anaerobic metabolism causes failure of ion-exchange channels, contributing to cell swelling and impaired enzymatic activity in the cytoplasm [8]. Subsequent to the initial stage of hypoxia, when blood supply is restored, the production of reactive oxygen species (ROS), nitrogen species, and the amplification of inflammatory responses, including the sequestration and activation of inflammatory cells, cytokine, and chemokines secretion, are locally increased, leading to secondary tissue detriment defined as IRI. The cell’s responsiveness to IRI depends on the intensity of the total tissue injury. Mild IRI may activate cell survival programs, whereas a moderate IRI may result in cell dysfunction and activate recovery systems for survival. In severe IRI-induced impairment, different mechanisms of cell death, such as apoptosis, necrosis, and autophagy, might be activated [9,10,11].

**Figure 1:**
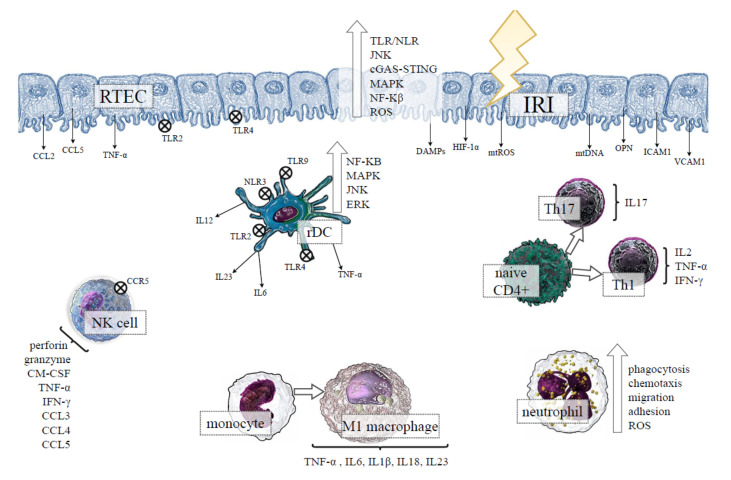
The effect of ischemia-reperfusion injury (IRI) on the renal tubular epithelial cells (RTECs) and the activation of innate immunity in kidney tissue. Under ischemic conditions, RTECs undergo injury and death, releasing diverse DAMPs, the products of oxidative stress, proinflammatory cytokines and chemokines, which can activate TLRs/NLRs, and distinct intracellular proinflammatory pathways in the neighbouring kidney cells along with the cells of the innate immune system. The results indicate the activation of rDCs, which intercommunicate with other cells of the immune system through proinflammatory cytokines secretion. After reperfusion, the recruitment of circulating monocytes, neutrophils, NK cells, and T cells occurs in the injured kidney. The proinflammatory environment favours the differentiation of macrophages into the inflammatory M1 phenotype, whereas naive CD4+ lymphocytes distinguish between the Th1 and Th17 proinflammatory subset, exacerbating kidney tissue damage. Abbreviations: TLRs—Toll-like receptor; NLR—NOD-like receptors; JNK–c-Jun NH(2)-terminal kinase; cGAS-STING—cyclic GMP-AMP and the cyclic GMP-AMP receptor stimulator of interferon genes; MAPK—mitogen-activated protein kinase; NF-κB—nuclear transcription factor-kappa B; ROS—reactive oxygen species; CCL—C-C motif chemokine ligand; CCR5—C-C motif chemokine receptor 5; TNF-α—tumour necrosis factor alpha; HIF-1α—hypoxia-inducible factor-1alpha; DAMPs—damage-associated molecular patterns; mtROS—mitochondrial reactive oxygen species; mtDNA—mitochondrial DNA; OPN—osteopontin; ICAM1—intercellular adhesion molecule 1; VCAM1—vascular cell adhesion molecule 1; ERK—extracellular signal-regulated kinase; IL—interleukin; rDC—renal dendritic cell; GM-CSF—granulocyte-macrophage colony-stimulating factor; IFN-γ—interferon gamma; Th—T helper cell.

A growing body of evidence supports the important role of pattern recognition receptors (PRRs) in developing AKI, such as Toll-like receptors (TLRs) and Nod-like receptors (NLRs) [12]. The above receptors are capable of being activated by the release of damage-associated molecular patterns (DAMPs) [13] or mitochondrial DNA (mtDNA) [14] from the injured renal tissue, and their activation triggers mechanisms of the innate immune response [15]. The renal tubular epithelial cells (RTECs), which constitute the majority of the cells in the kidney, can also contribute to innate immunity, since they function as physical barriers that are able to recognise danger signals and produce proinflammatory cytokines and chemokines [16]. Moreover, RTECs can cooperate with diverse elements of the immune system during IRI, amplifying the inflammatory response, as presented in Figure 1. Although the inflammatory response is needed for proper tissue repair and regeneration after IRI, the excessive inflammation may result in renal tissue damage, fibrosis and, ultimately, the loss of kidney function.

In the present review, we discuss the role of indoleamine 2,3-dioxygenase (IDO1), a tryptophan metabolizing enzyme with immunoregulatory function [17], as a factor modulating the activity of the innate immune system in the course of IRI-induced AKI.

## 2. Role of Renal Tubular Epithelial Cells (RTECs) in IRI-Induced AKI

RTECs represent 75% of the parenchymal cells of the kidney and are able to express varied proinflammatory molecules: tumour necrosis factor α (TNF-α), transforming growth factor β (TGF-β), Fas/Fas ligand (FasL), and adhesion molecules—intercellular adhesion molecule 1 (ICAM-1) and vascular cell adhesion molecule-1 (VCAM-1), whose production is particularly amplified in response to ischemia and oxidative stress [18,19,20]. The disruption of homeostasis between oxygen supply and demand during ischemia causes a reduction in oxidative metabolism with progressive injury and death of RTECs. It is believed that necrosis, as well as apoptosis of these cells, is a prominent feature of renal IRI [21,22].

The hypoxic and anoxic cell injuries supervene in the microvasculature and tubular epithelium [23], leading to the production of endogenous TLR and NLR ligands. One of the earliest events in the development of IRI is the nonbacterial (sterile) activation of innate immune receptors such as TLRs and NLRs, which are both expressed in renal cells as well as in resident immune cells. The aforementioned structures can recognise DAMPs and trigger several intracellular pathways, such as nuclear factor ĸB (NF-ĸB), c-Jun N-terminal kinases (JNK), and mitogen-activated protein kinase (MAPK), which are associated with the secretion of proinflammatory cytokines and chemokines [12,13,24]. Various TLRs are expressed in renal compartments and are capable of contributing to the immune system inflammatory responses. No expression of TLR2 or TLR4 was proven in the renal tissue of healthy rodents; however, in IRI conditions, RTECs become necrotic and released DAMPs, which triggered TLR2/4 on the adjoining renal cells [25]. Furthermore, ischemia-induced renal inflammation, mediated by TNF-α and interferon γ (IFN-γ), significantly enhanced TLR2 and TLR4 expression in the Henle’s loop and collecting ducts [26]. The current knowledge regarding the role of TLRs in AKI proceeded from studies of TLR4 and/or TLR2 in knockout mice, which were protected from IRI-induced AKI. Importantly, it has been shown that TLR2 signalling in RTECs is an initial mechanism to remove injured cells by inducing acute inflammation following kidney IRI [27,28]. Upon exposure to TLR2/4 ligands, RTECs produce proinflammatory cytokines and chemokines such as CCL2/MCP-1 and CCL5/RANTES, which are the major chemoattractants participating in the recruitment of immune cells to the harmed tubular epithelium [24].

In contrast to ischemia, reperfusion restores aerobic metabolism yet concomitantly induces the overproduction of ROS, causing direct oxidative damage of all biological molecules including mitochondrial proteins, lipids, and genomic and mitochondrial DNA. The above processes result in impaired mitochondrial bioenergetics and an increase in their membrane permeability [29]. As a consequence of leakage into the cytosol, mitochondrial DNA (mtDNA) have the ability to activate the cyclic GMP-AMP synthase (cGAS), the stimulator of interferon genes (STING) pathway (cGAS-STING), initiating the transcription of innate immunity-related genes, tubular inflammation, and AKI progression. Genetic depletion of STING significantly improved the neutrophils count, production of proinflammatory cytokines and chemokines, and diminished tubular inflammation [14]. mtDNA manages to be also recognised by TLR9; thus, it may contribute to IRI-induced AKI [30], and the oxidised form of DNA is probably a more powerful stimulator than the nonoxidised form of DNA [31]. Moreover, mitochondrial ROS (mtROS), themselves, are capable of inducing renal injury by activating proinflammatory signals such as TLRs and the NLRP3 inflammasomes [32]. All of the above mechanisms contribute to RTECs death, mainly due to the fact of apoptosis and ferroptosis as has been demonstrated in human RTECs cultured in anoxic conditions and pursuant to the reoxygenation [22].

## 3. Innate Immunity in IRI-Induced AKI

Despite the diverse aetiologies underlying AKI, the immune system is an important determinant in the initiation of most forms of kidney injury. The immune system consists of innate and adaptive immunity, whose main function is to effectively combat pathogens that threaten the body; nonetheless, it also performs an important role in maintaining balance and not leading to overactivation, generating the occurrence of autoimmune disorders [33].

The complex defence mechanisms are activated regardless of the type of the danger factor; therefore, innate immunity is considered a nonspecific immune response and is the first, direct, and immediate line of defence against pathogens or endogenous molecules, which are recognised as “nonself” danger signals (DAMPs) [34].

Innate immunity can be divided into cellular and noncellular sections, containing dendritic cells (DCs), eosinophils, monocytes/macrophages, neutrophils, a specific population of lymphocytes—natural killer (NK) cells—and several other immune cell types that have the ability to operate through various cytokines and activate the complement cascade, leading to activation of the adaptive immune system [35].

### 3.1. Role of Kidney Resident Immune Cells in IRI-Induced AKI

Different resident immune cells belonging to innate immunity, such as antigen-presenting DCs, phagocytic macrophages, and natural killer (NK) cells, are located in the kidney. The abovementioned cells, as well as epithelial and endothelial cells, are in close connection with parenchymal cells, playing an important role in the maintenance of tissue homeostasis [25,36].

Resident renal DCs (rDCs) are the dominant leukocyte subset in the kidney [25], which in healthy conditions present an immature phenotype [37]. During IRI, rDCs maturation is mediated by hypoxia-inducible factor 1α (HIF-1α) [38] and proinflammatory substances released by hypoxic tubular cells [39,40]. Following maturation, rDCs present high surface levels of the histocompatibility complex class II (MHC II) and costimulatory ligands—CD80/CD86. In addition, they possess potent T-cell stimulatory ability—they are capable of presenting self-antigens to CD8+ T cells, NKT cells, or antigen-specific CD4+ T cells within draining renal lymph nodes [37,41,42]. The interactions between antigen-presenting DCs and T-cell subsets can be injurious to the kidney, as the blockade of the CD80/CD86 costimulatory ligands [43] or depletion of rDCs by clodronate [44] attenuate IRI-induced AKI.

rDCs secrete TNF-α, which is essential to neutrophil influx post-IRI and RTECs apoptosis [45,46]. Moreover, the activated rDCs can emit proinflammatory cytokines, such as IL-1, IL-6, IL-12, and IL-23, which intensify inflammatory response [47]. Rama et al. [48] showed that kidneys exposed to ischemia demonstrated an increase in the number of viable mature rDCs, which may respond to endogenous activators of the innate immunity, provoke the secondary responses in RTECs and endothelial cells, as well as participate in the recruitment of additional circulating cells into the kidney. They also established that HIF-1 target gene activation was linked with DCs’ maturation under hypoxic conditions and that the presence of mechanistic target of rapamycin (mTOR)-dependent signalling facilitated the expression of hypoxia-induced genes. The aforementioned findings suggest that rDCs have a proinflammatory role in IRI; however, several studies revealed that these cells can also prevent ischemic tissue damage in the mechanism of anti-inflammatory signalling through interferon regulatory factor 4 (IRF4), IL-10, and a single Ig IL-1-related receptor [49,50].

Residual macrophages are a second type of cell that constitute an innate arm of the immune system in the kidney. Macrophages play a major role in the maintenance of tissue homeostasis and integrity—they recognise the initial damage signals released from dying renal cells and participate in the elimination of senescent and dead cells, extracellular matrix remodelling, antigen presentation, and TNF-α production. However, macrophages also take part in the restoration of lost cells through the production of regenerative growth factors [51].

The innate immune system is activated ahead of an adaptive response. After the initial injury response, resident macrophages, rDCs, and hypoxic endothelial cells prolong inflammation further, expressing chemoattractant chemokines and cytokines, which cause an influx of other circulating leukocyte subsets, e.g., neutrophils, monocytes/macrophages, and lymphocytes into the site of injury [37,51,52,53,54]. These infiltrating cells directly contribute to kidney parenchymal damage and may exacerbate IRI [55,56]. It has been proved that depletion of macrophages [44,57], DCs [44], and neutrophils [58] protect kidney tissue from IRI.

Activated DCs and macrophages can also present the renal-derived antigens to effector T cells, which rapidly infiltrate the kidney after reperfusion, being effective in the exacerbating inflammatory response. Secondary tissue injury is raised as the result of the above mechanism [59]. However, the other subset of T cells—T regulatory cells (Tregs)—have been proven to suppress the extent of kidney IRI through an IL-10-dependent mechanism [60,61].

### 3.2. Macrophages in IRI-Induced AKI

Apart from kidney resident macrophages that are able to locally proliferate and self-renew [62], the other cluster of macrophages (i.e., monocyte-derived macrophages) is rapidly recruited into injured kidneys during IRI, and then differentiate into distinct macrophage phenotypes, depending on the cytokine environment or interactions with other cells [63]. Li et al. [64] showed that the following ischemia and after 3 h of reperfusion, the inflamed monocytes secreting IL-6, TNF-α, IL-1α, IL-12, and expressing C–C motif chemokine receptor 2 (CCR2) and C-X3-C motif chemokine receptor 1 (CX3CR1) appeared in injured kidneys. Both CCR2 and CX3CR1 were necessary for the migration of monocytes, because depletion of these receptors caused reduction of the infiltrated cells and protection of CCR2- and CX3CR1-knockout animals from IRI-induced AKI.

Macrophages have the ability to actively participate in all IRI-related processes, including cell destruction and the regeneration phase, and their phenotypes can change, depending on the IRI stage. Generally, M1 macrophages are considered as proinflammatory, which are able to secrete TNF-α, IL-6, IL-1β, IL-18, and IL-23 [65,66], while M2 macrophages maintain immunomodulatory functions, wound repairing abilities, and tissue remodelling capacity, along with producing diverse cytokine and chemokine profiles such as IL-4, TGF-β1, IL-10, CCL18, and CCL22 [65,67].

M1 macrophages are the first responders to injury—they produce proinflammatory cytokines, whose expression appears within the first 24 h post-IRI and decreases at 3 days post-injury [68]. They also secrete the cytotoxic compounds: nitric oxide synthase (NOS) and ROS, which induce mitochondrial damage and apoptosis [51]. It has been shown that prolonged activation of M1 macrophages harmed kidney recovery after IRI. In contrast, the appearance of M2 macrophages correlated with the proliferative phase of kidney repair [69,70]. Zhang et al. [71] demonstrated that effective polarisation of macrophages to the M2 phenotype, which is associated with wound healing and tissue repair, is essential for recovery from AKI. The above results suggest that the initial inflammatory response of M1 macrophages is necessary for the clearance of damaged and pathogenic particles, whereas a prolonged inflammatory state contributes to further tissue damage.

Data concerning human macrophage phenotypes and related cytokines in AKI kidneys are scarce, limiting the translation of preclinical studies to humans. Nevertheless, in biopsies of kidneys from patients with underlying AKI, macrophages have been identified as the main infiltrating cell type in the kidney that persists during tissue repair [72,73]. The abovementioned macrophages undergo a partial differentiation to the M2 phenotype characterised by CD163 overexpression and IL-10 release [74]. Studies performed in vitro on human macrophages proved that CD163 overexpression induced a change in the cytokine profile secretion from M1-related proinflammatory to M2-related anti-inflammatory cytokines [75]. Moreover, depletion of macrophages before IRI improved kidney function and markers of tubular injury in the histological examination, whereas reduction of cells during the reparative phase resulted in an intensification of injury markers [76]. Thus, the functional switch in the macrophage phenotype controls the long-term outcomes upon AKI, and an anti-inflammatory M2 phenotype succours the resolution of post-ischemic inflammation and promotes wound healing, reversing IRI-induced AKI [77].

### 3.3. Neutrophils in IRI-Induced AKI

As a result of ischemic IRI, only 30 min after the reperfusion, neutrophils adhere to the endothelium in peritubular capillaries and gather in the kidney interstitium in animal models as well as in human AKI [78]. Soon after reaching the renal parenchyma, neutrophils are exposed to DAMPs, which leads to activation of TLRs and cytotoxicity via phagocytosis, chemotaxis, and oxidative burst [79].

Data from the literature on the role of neutrophils in the development of IRI-induced AKI are inconclusive. The first study by Thornton et al. [80] showed that the depletion of neutrophils in rats before IRI induction conferred neither functional nor morphologic protection when compared with the controls. However, further studies performed on the IRI-induced AKI model in rats demonstrated that neutrophil depletion or blocking of their activation may prevent renal dysfunction, confirming a contribution of these cells to AKI [58,81,82].

Neutrophils release cytotoxic histones undergoing neutrophil extracellular trap (NET) formation, which are able to contribute to AKI severity. Nakazawa et al. [83] demonstrated the NET formation in kidney biopsies from patients with post-transplant acute tubular necrosis caused by long cold ischemia as well as in the post-ischemic kidney of mice. Pretreatment with inhibitors of NET or anti-histone IgG suppressed ischemia-induced NET formation and renal injury. Importantly, they also showed that ischemic injury observed in the kidney was associated with increased levels of circulating histones and neutrophil infiltration in distant organs, such as lungs, and inhibition of NET formation or anti-histone therapy can also prevent remote organ injury [83]. Similarly, it has been shown that depletion of neutrophil enzyme—peptidyl arginine deiminase-4 (PAD4), which is involved in the NET formation—can partially preserve the kidney function in mice subjected to IRI [84].

The above data suggest that neutrophil-mediated tubular necrosis, histone release, and NET formation not only exacerbate kidney injury but may also lead to multiorgan dysfunction.

### 3.4. Natural Killer (NK) Cells in IRI-Induced AKI

NK cells are critical to the innate immune system, as they have the ability to recognise injured cells in the absence of a major histocompatibility complex [85]. NK cells infiltrate the kidney soon after IRI (after 3 h of renal IRI), and they correlate with serum creatinine levels. The count of NK cells decreased significantly 24 h following renal IRI [86].

NK cells are cytotoxic lymphocytes that have an important role in IRI. The direct cytotoxic effect of these cells is mainly accomplished by two pathways: the induction of apoptosis of target cell by secretion of membrane-disrupting proteins and proteases or by caspase-dependent apoptosis involving the death receptors on target cells [87]. They also contain perforin and granzymes, which are released in proximity to target cells, causing apoptosis or osmotic cell lysis [85].

Besides their direct cytotoxicity, NK cells release various cytokines and chemokines such as GM-CSF, IFN-γ, TNF-α, CCL3, CCL4, and CCL5 [88]. These cells constitutively express receptor CCR5 for the above molecules, which functions as the major receptor for NK cells during kidney IRI. The blocking of CCR5 completely inhibited NK cell migration in vitro and in vivo [89]. NK cell and neutrophil recruitment by RTECs during IRI was sequential and independent—the neutrophil migration prior to NK cell infiltration was needed [89]. It has also been shown that NK cells can directly kill RTECs, contributing substantially to kidney IRI, and NK cell depletion in wild-type C57BL/6 mice was protective in kidney IRI [90].

Several factors have been identified as regulators of NK cell activation/infiltration during IRI. Zhang et al. [90] found that NK cells can induce RTECs death through interaction between the NK group 2 member D (NKG2D) receptor present on their surface and its ligand—retinoic acid early induced transcript-1 (Rae-1). As a result of renal damage, osteopontin (OPN) expression was significantly upregulated in all tubular segments and glomeruli [91]. Zhang et al. [92] used OPN knockout mice to show that OPN promoted NK cell-induced renal damage during IRI. They also demonstrated in vitro conditions that OPN has the ability to cause NK cell migration and activation, promoting RTEC death. Furthermore, OPN can activate NK cells to express granzyme B, TNF-α, IFN-γ, and activated NK cells may induce apoptotic death in RTECs [93]. On the other hand, a study by Noiri et al. [94] found that disruption of the OPN gene in mice kidneys resulted in increased injury after IRI, suggesting a renoprotective role of OPN. The above discrepancy was settled by Cen et al. [95], who used anti-OPN antibodies in mice subjected to IRI and observed that IL-6 and TNF-α levels were reduced, NK cells and neutrophils infiltration was decreased, whereas the histologic architecture and apoptosis of renal tissue were improved in these animals. Their findings confirmed the harmful capacity of OPN in NK-mediated renal injury during IRI.

In conclusion, NK cells play an essential role in the pathogenic axis linking RTECs and neutrophils in kidney IRI.

## 4. IDO1 and Innate Immunity in IRI-Induced AKI

Indoleamine 2,3 dioxygenase 1 (IDO1) is a rate-limiting enzyme with immunomodulatory properties, which degrades tryptophan (TRP) through the kynurenine pathway (KP) [96]. It is well documented that IDO1 expression may be induced in various cells of innate immunity, including DCs, macrophages, NK cells, and neutrophils in response to the proinflammatory microenvironment [97,98,99]. The role of IDO1/KP activation in the regulation of immune responses has been recently widely described by us [100]. Generally, IDO1 may initiate two main pathways: (1) by TRP depletion, it activates the general control non-derepressible 2 kinase (GCN2K) pathway and inhibits the mammalian target of rapamycin (mTOR) signalling, leading to T-cell autophagy, anergy, and apoptosis; (2) the produced kynurenine (KYN) and its metabolites can activate the aryl hydrocarbon receptor (AhR), contributing to the long-lasting immunotolerance by affecting the balance of the Th1/Th2 and Th17/Tregs systems [100].

### 4.1. IDO1 Expression by RTECs in IRI-Induced AKI

Though the presence of the active kynurenine pathway has been previously observed in the kidney tissue of rats with chronic renal failure [101], Mohib et al. [102] showed, for the first time, that functional IDO1 is expressed by RTECs in vitro and is rapidly increased after exposure to IFN-γ and TNF-α. Increased IDO1 activity can regulate caspase-8 activation and RTECs apoptosis via a Fas/FasL-dependent mechanism. Moreover, a potentiating effect of cytokine-mediated RTEC death upon exposure to downstream KP metabolites: picolinic acid (PA) and quinolinic acid (QUIN) has been demonstrated. The inhibition of IDO1 by the specific inhibitor 1-methyl-d-tryptophan (1-MT), blocking caspase-8 or use of FasL-deficient RTECs line attenuated these cells’ apoptosis, indicating a pivotal role of the IDO1/Fas/FasL/caspase-8 axis in apoptotic death of RTECs. The above data suggest that renal IDO1 expression and accumulation of KP metabolites may be potentially harmful during renal inflammation accompanying IRI. This hypothesis was subsequently confirmed by this same team in vivo, using a mouse model of IRI [58]. IDO1 mRNA expression and protein levels increased at 2 h post-reperfusion and remained elevated for up to 24 h compared to the control animals. Interestingly, it was observed that enhanced IDO1 expression in RTECs was related to the reperfusion-associated inflammatory cells influx and their cytokines rather than to ischemia alone. The data suggested RTECs appear to be primarily responsible for the elevated IDO1 expression during IRI. Furthermore, both IDO1 inhibition and its deletion resulted in improvement of kidney function and reduced numbers of RTECs undergoing apoptosis or necrosis. Based on these observations, Mohib et al. [58] concluded that therapeutic inhibition of IDO1 may be beneficial in the early stages of transplant injury that involve IRI, especially while RTECs express high IDO1 levels.

**Figure 2:**
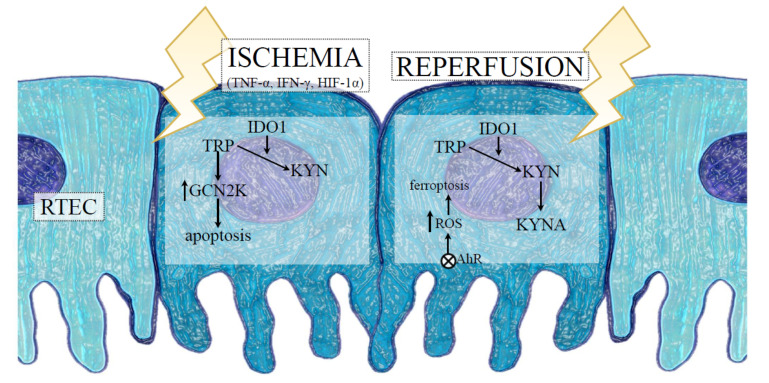
The role of indoleamine 2,3 dioxygenase 1 (IDO1) in RTEC death during IRI-induced AKI. The hypoxic conditions and proinflammatory cytokines released into the intrarenal environment, following the ischemic phase of IRI, induce IDO1 expression and KYN synthesis with simultaneous depletion of TRP in RTECs. The reduction of TRP in the microenvironment results in activation of GCN2K and apoptosis-mediated death of RTECs. After reoxygenation, IDO1-dependent activation of the kynurenine pathway in addition to the production of KYN and KYNA activates AhR, which triggers ROS generation, leading to AhR-mediated ferroptosis of RTECs. Abbreviations: RTECs—renal tubular epithelial cells; IRI—ischemia-reperfusion injury; TNF-α—tumour necrosis factor alpha; IFN-γ—interferon gamma; HIF-1α—hypoxia-inducible factor-1alpha; TRP—tryptophan; KYN—kynurenine; KYNA—kynurenic acid; GCN2K—general control nonderepressible 2 kinase; ROS—reactive oxygen species; AhR—aryl hydrocarbon receptor.

The aforementioned finding complies with a clinical study showing the presence of high KYN concentrations in serum and urine of kidney recipients during acute rejection, suggesting that IDO1-dependent KP activation may participate in graft injury [103]. Eleftheriadis et al. [104] demonstrated that in cultures of proximal RTECs surrounded by reoxygenation, the ferroptotic death of cells depended on the activation of AhR. Since KYN and a certain amount of downstream products of its metabolism, such as kynurenic acid (KYNA), are well known endogenous activators of AhR [105], it has been suspected that the reoxygenation-induced AhR activation could result from the IDO1 upregulation and the subsequent kynurenines overproduction. A recent study by Eleftheriadis et al. [106] confirmed the presented hypothesis, demonstrated that IDO1 was upregulated during anoxia and induced GCN2K-mediated apoptosis in RTECs, whereas the reoxygenation also upregulated IDO1, increased KYN levels, and triggered AhR-induced ROS generation, leading to AhR-mediated ferroptosis of these cells (Figure 2). In addition, the inhibition of IDO1 prevented both pathways, counteracting the damage of RTECs. These data, although limited only by the in vitro conditions, suggest that KP activation occurs in either phase of IRI and can contribute to kidney injury as a result of IRI.

A recent study by Pan et al. [107] demonstrated, in vivo, that in a mouse model of IRI-induced AKI, the expression of IDO1 in renal tissue was increased at day 14 after IRI, and it was related to activation of the Wnt/β-catenin pathway, kidney fibrosis, and impaired kidney function. In contrast, kidney fibrosis was lower, although kidney function was better in IDO1-knockout animals compared to WT animals in this experimental IRI model.

**Figure 3:**
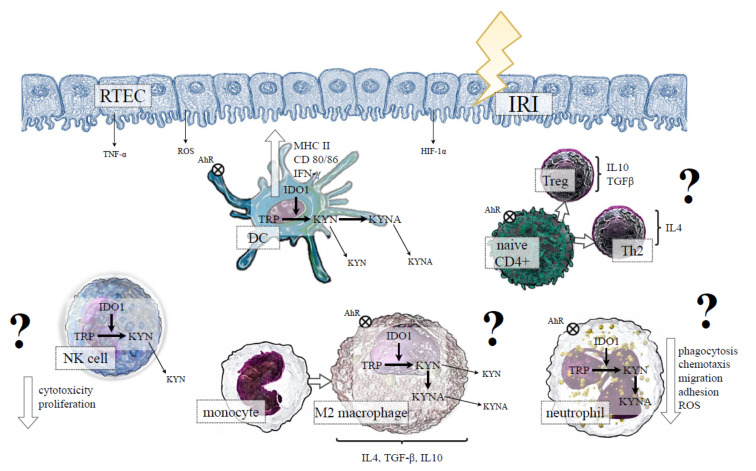
The hypothetical role of indoleamine 2,3 dioxygenase 1 (IDO1) in the cells of innate immunity during IRI-induced AKI. IRI-induced hypoxia, proinflammatory conditions and oxidative stress initiate maturation of DCs, which is associated with the activation of IDO1 and the production of KYN and its downstream metabolites. Kynurenines, through AhR activation, determine a tolerogenic DC phenotype, which can potentially promote naive CD4+ cells differentiation into anti-inflammatory Th2 and Treg subsets. The expression of IDO1 in macrophages may be potentially associated with M2 phenotype polarisation. In neutrophils, IDO1 activation and KYN production could result in reduced phagocytosis, ROS production, and migration, whereas IDO1 induction in NK cells may potentially reduce cytotoxicity and proliferation of these cells. All above mechanisms, through amelioration of kidney damage, could be beneficial in IRI-induced AKI (?); however, at the moment, no data confirm the presented hypothesis. Abbreviations: RTEC—renal tubular epithelial cells; IRI—ischemia-reperfusion injury; TNF-α—tumour necrosis factor alpha; HIF-1α—hypoxia-inducible factor-1alpha; ROS—reactive oxygen species; MHC II—major histocompatibility complex class II; IFN-γ—interferon gamma; TRP—tryptopha; KYN—kynurenine; KYNA—kynurenic acid; AhR—aryl hydrocarbon receptor; DC—dendritic cell; NK—natural killer; IL—interleukin; TGF-β—transforming growth factor beta; Treg—regulatory T cell; Th—T helper cell.

As opposed to the above, a study by Wan et al. [108] showed that the expression of IDO1 in RTECs was consistent with the infiltration of Tregs producing IL-10, increased proliferation of the surviving RTECs, and kidney recovery after IRI in a mouse model of AKI. Thus, it is possible that IDO1 could play a dual function in the inflammatory response during IRI, depending on the timing of ischemia and the environmental conditions [109]. The situation becomes more complicated if the influence of the immune cells that can express IDO1 in the course of IRI is considered as is presented in Figure 3.

### 4.2. IDO1 and DCs in IRI-Induced AKI

It has been shown that IDO1 activity increased during DCs maturation, and it was related to phenotypic and functional changes of DCs, responsible for generating MHC/peptide complexes and priming T cells [110]. The abovementioned suggests that IDO1-expressing DCs can have a proinflammatory role in IRI. The contemporary study of Čepcová et al. [111] showed the inhibition of IDO1 by two isomers: 1-methyl-d-tryptophan (D-MT) and 1-methyl-l-tryptophan (L-MT), which decreased plasma KYN, improved kidney function after IRI, with D-MT having a faster action though shorter duration than L-MT. Moreover, L-MT reduced 3-HKYN levels and increased the overall survival of these rats. In IRI-induced DCs, the treatment with L-MT decreased the IDO1 protein level, whereas D-MT had no effect. Notwithstanding, T cells co-cultured with IRI-induced DCs treated with both isomers exhibited a phenotype steered towards renoprotective Th2. At the same time, IRI-induced DCs treated with L-MT caused a reduction in FoxP3 expression, being a key transcription factor for tolerogenic Tregs. In general, a decreased proliferation of T cells stimulated with IRI-induced DCs and treated with both MT isomers was observed. Furthermore, both isomers decreased the expression of TLR4 signalling in DCs [111]. The indicated study demonstrates that the effect of DCs on the IRI course can be either IDO1-dependent or -independent, involving mutually separate mechanisms such as the downregulation of TLR4 and its signalling molecules.

A few pieces of research indicate that DCs can also have a protective role in ischemic kidney injury [50]. In addition, it was proven that the intracellular upregulation of IDO1 in DCs was able to induce immunotolerance [112]. KYN and its metabolite—KYNA—are endogenous ligands for AhR on DCs. Furthermore, the AhR activation by the kynurenines may determine a tolerogenic DCs phenotype, promoting Tregs expansion [106,113]. Thus, IDO1 expression in DCs is likely to modulate the immune response of effector cells. The posed hypothesis was confirmed in numerous studies in which IDO1-expressing DCs formed part of the mechanism limiting excessive activation of the immune system through the reduction of effector T-cell proliferation and induction of their apoptosis [112,114], promotion of Tregs differentiation [115], or favouritism of the polarisation of proinflammatory Th1 and Th17 subsets into tolerogenic Th2 and Tregs [116]. Contrariwise, depletion of IDO1-expressing DCs resulted in increased T-cell proliferation and intensification of inflammation [117].

Apart from the described above role of AhR in the induction of immunotolerance, the activation of this receptor can act antagonistically towards the HIF-1α subunit [118], and IDO1 may exert a further renoprotective effect mediated by the kynurenines/AhR/HIF-1α axis.

### 4.3. IDO1 and Macrophages in IRI-Induced AKI

Macrophages can express IDO1 after stimulation with INF-γ [119]. Additionally, it is associated with the switching of the proinflammatory M1 to the tolerogenic M2 phenotype, whereas the silence of IDO1 induces the formation of M1-type macrophages [98]. It has been shown that IDO1 expression in macrophages is substantial for keeping the balance between anti- and proinflammatory effects of these cells in a mouse model of *Aspergillus fumigatus* keratitis [120]. The indicated diverse role of macrophages in the inflammatory responses may be partly dependent on the presence of AhR, as AhR-deficient macrophages showed a higher level of proinflammatory cytokines upon LPS stimulation, and AhR-deficient mice were more susceptible to LPS-induced lethal shock than control animals [121]. Recently, elevated IDO1 activity is considered a feature of M2 macrophage activation [122].

Although a direct effect of IDO1 on macrophage function and polarisation during IRI-dependent AKI is yet unknown, a recent study by Li et al. [123] described IDO1-dependent macrophage polarisation in a mouse model of myocardial ischemia-reperfusion. In in vitro conditions, p-coumaric acid-induced M2 macrophage polarisation through an IDO1-dependent mechanism occurred, whereas the M1 subpopulation of macrophages was reduced. In vivo, IDO1 expression was downregulated in mouse hearts subjected to myocardial IRI. The treatment using p-coumaric acid increased IDO1 expression in the hearts of IRI mice and decreased inflammation around heart tissue, apoptosis of cardiomyocytes caused by IRI, and the number of M1 macrophages [123]. The above results suggest that the promotion of M2 macrophage polarisation through IDO1 may attenuate macrophage-mediated inflammation following myocardial IRI. However, further research is required to determine whether a mechanism of that kind would also occur in IRI-induced AKI.

### 4.4. IDO1 and Neutrophils in IRI-Induced AKI

The human *Chlamydia*-infected neutrophils were able to express the IDO1 gene and enzymatic activity after induction with IFN-γ; moreover, downstream KP metabolites, such as KYNA, 3-hydroxyanthranilic acid (3-HAA), PA, and QUIN, were also detected in the *Chlamydia*-infected and IFN-γ-treated cells. However, chlamydial growth in HL-60 neutrophils was not influenced by the addition of IFN-γ, suggesting that these cells might serve as a refuge for *Chlamydia* in an IFN-γ -rich microenvironment [124]. Loughman et al. [125] observed that uropathogenic *Escherichia coli* infection attenuated an innate response by inducing IDO1 expression in human neutrophils in vitro through the reduction of phagocytosis, decreasing the production of antimicrobial ROS, downregulating their chemotaxis, adhesion, and migration. In contrast, the treatment toward neutrophils with IDO1 inhibitor significantly enhanced their transepithelial migration in response to uropathogenic *Escherichia coli*. Further, this same team explained that neutrophil migration was suppressed by an IDO1-mediated increase in the local production of kynurenines [126]. Similarly, Tregs may play an important role in the direct control of the innate immune response based on the induction of immunosuppressive neutrophils producing IDO1 [127]. The results indicate that the induction of IDO1 expression in neutrophils can mitigate proinflammatory innate responses, as neutrophil function was not affected in IDO1-knockout mice [128]. Nevertheless, the existing literature lacks data on the role of IDO1-expressing neutrophils in innate immunity during IRI-induced AKI.

### 4.5. IDO1 and NK Cells in IRI-Induced AKI

Currently, there are no data on how the induction of IDO1 in NK cells translates into the course of ischemia-induced AKI. The studies performed on NK cells in cancers showed that INF-γ can induce IDO1 mRNA expression in NK cells, and pharmacological inhibition of the enzyme may reduce cytotoxicity against cancer cells both in in vitro [129] and in vivo conditions [99]. Thus, the induction of IDO1 in NK cells could maintain the standard cytotoxicity against tumour cells. On the other hand, it has been reported that kynurenines—the catabolites of IDO1—can block the proliferation of NK cells [130] by downregulation of their natural cytotoxic receptors, NKp46 and NKG2D, suppressing their cytolytic activity and inducing NK cell death [131]. Moreover, it has been demonstrated that KYN treatment is able to regulate NK cells via STATs signalling pathways, blocking of IDO1 activity in tumour cells, restored NK cells’ cytolytic activity and receptors expression [131].

Theoretically, it seems to be possible that induction of IDO1 in NK cells during kidney IRI may favour its cytotoxic activity, while the expression of IDO1 and elevated production of KYN through the cells present in the surrounding microenvironment can limit the immune function of NK cells, reducing cellular damage to kidney tissue. This hypothesis complies with the research of Masoumy et al. [112], who demonstrated that without IDO1 present in the environment, inflammation induced by the IRI in the hind limb was more pronounced, resulting in necrosis and apoptosis of the tissues, which led to a longer recovery time seen clinically. However, whether such interactions can occur under hypoxic conditions of AKI requires further investigation.

## 5. Conclusions and Future Perspectives

IDO1 is an important moderator of the innate immune system with the ability to modify tolerance and suppression depending on environmental factors. APCs, such as DCs or macrophages, possess the complete set of KP enzymes, indicating the functional role of the KP in immune cells’ biology. However, it remains to be established whether the status of immune cells concerning IDO1 is a determinant of the local inflammatory response or if it reflects a compensatory response aimed at the prevention of kidney tissue damage in the surroundings of IRI. The paucity of literature data in this field does not allow for an unambiguous answer at this moment. Nevertheless, limited data have indicated that the mitigation of the excessive innate immune response may improve the clinical care for patients with IRI-induced AKI.

Due to an increased global prevalence rate, IRI-induced AKI is directly responsible for adverse clinical outcomes and high health care costs. Thus, it is clinically relevant to have a comprehensive understanding of the innate immune system, in particular the pivotal mediators and immune pathways that contribute to the pathogenesis of IRI-induced AKI.

Discovering the mechanisms of crosstalk between different intra- and intercellular pathways during inflammatory conditions, such as IRI, and relating them to new ideas, and findings such as IDO1 will allow to more appropriately and effectively apply the aforementioned concepts towards new therapies.

We hope that the presented review may offer new research directions for the development of combining therapies, which will allow for the proper regulation of the IDO1-dependent function of the innate immune response in the course of IRI-induced AKI.

## Data Availability

Not applicable.
